# Evolving Longitudinal Retinal Observations in a Cohort of Survivors of Ebola Virus Disease

**DOI:** 10.1001/jamaophthalmol.2020.0173

**Published:** 2020-03-05

**Authors:** Paul J. Steptoe, Fayiah Momorie, Alimamy D. Fornah, Patrick Komba, Elizabeth Emsley, Janet T. Scott, Samantha J. Williams, Simon P. Harding, Matthew J. Vandy, Foday Sahr, Nicholas A. V. Beare, Malcolm G. Semple

**Affiliations:** 1 Department of Women’s and Children’s Health, Faculty of Health and Life Sciences, University of Liverpool, Liverpool, England; 2 St Paul’s Eye Unit, Royal Liverpool University Hospital, Liverpool, England; 3 National Institute for Health Research Health Protection Research Unit in Emerging and Zoonotic Infections, University of Liverpool, Liverpool, England; 4 Medical Research Council, University of Glasgow Centre for Virus Research, Glasgow, Scotland; 5 34 Military Hospital, Freetown, Sierra Leone; 6 Department of Eye and Vision Science, Faculty of Health and Life Sciences, University of Liverpool, Liverpool, England; 7 Connaught Hospital, Freetown, Sierra Leone

## Abstract

**Importance:**

The 2-year ophthalmic sequelae of Ebola virus disease (EVD) in survivors of the 2013 to 2016 epidemic is unknown and may have public health implications for future outbreaks.

**Objective:**

To assess the potential for uveitis recurrence, the behavior of dark without pressure, and visual outcomes in a cohort of Sierra Leonean survivors of EVD 2 years following the 2013 to 2016 Ebola epidemic.

**Design, Setting, and Participants:**

Prospective, 1-year observational cohort study performed between 2016 and 2017 at 34 Military Hospital, Freetown, Sierra Leone. Participants included survivors of EVD who reported ocular symptoms since Ebola treatment unit discharge and were participants of a previous case-control study. Participants were invited for ophthalmic reexamination and finger-prick blood sampling for immunoglobulin G (IgG) to *Toxoplasma gondii* and HIV.

**Exposures:**

Ebola virus disease.

**Main Outcomes and Measures:**

Primary outcome measure: comparative ultra-widefield retinal imaging. Secondary outcome measures: visual acuity and detection of IgG to *T gondii *and HIV.

**Results:**

Of 57 survivors of EVD who underwent repeated ophthalmic evaluation, 37 were women (64.9%). Mean (SD) age was 31.9 (11.1) years. Median interval between first and last examination was 370 days (interquartile range [IQR], 365-397.5 days), and median time from discharge to last examination was 779 days (IQR, 732-821 days). Fifteen eyes of 10 survivors (17.5%) had retinal lesions secondary to EVD. No new EVD-associated retinal lesions were observed. Two survivors (3.5%) developed new posterior uveitis resembling toxoplasmosis chorioretinitis and 41 (73%) were seropositive for *T gondii* IgG. Areas of dark without pressure were observed either confined to the perimeter of Ebola retinal lesions (n = 7) and non-Ebola lesions (n = 2), involving extensive retinal areas adjacent to Ebola retinal lesions (n = 4) and non-Ebola lesions (n = 2) or in isolation (n = 6). Both expansion and regression of areas of dark without pressure were observed over the study period. Best eye–presenting visual acuity had mild or no visual impairment in 55 survivors (96.4%) 2 years following discharge.

**Conclusions and Relevance:**

Vision was maintained in survivors of EVD 2 years following discharge. Evolving regions of dark without pressure may be associated with EVD retinal lesions and might suggest the presence of an ongoing intraretinal stimulus, which may be associated with infective etiology. Treatment strategies should account for the possibility of toxoplasmosis chorioretinitis recurrence within survivors of EVD.

## Key Points

**Question:**

What are the ophthalmic sequelae in survivors of the 2013 to 2016 Ebola epidemic?

**Findings:**

In this cohort study, no new Ebola retinal lesions were observed and visual acuity was preserved at 2 years; however, associated retinal dark without pressure did change with regression and expansion of affected zones. New clinical toxoplasmosis chorioretinal lesions occurred in 2 survivors.

**Meaning:**

Expanding dark without pressure might suggest an ongoing intraretinal stimulus, which may be associated with a viral infection; treatment strategies might take account of the possibility of toxoplasmosis chorioretinitis recurrence within survivors of Ebola.

## Teaser

This cohort study assesses the potential for uveitis recurrence, the behavior of dark without pressure, and visual outcomes in a cohort of Sierra Leonean survivors of Ebola virus disease 2 years following the 2013 to 2016 Ebola epidemic.

## Introduction

Ebola virus remains a serious public health threat. Research conducted following the aftermath of the 2013 to 2016 West African Ebola virus disease (EVD) epidemic reported the prevalence of uveitis within survivors during the convalescent period was between 13% and 34%.^1,2,3^ The aqueous humor of 1 repatriated US survivor identified viable Ebola virus during active uveitis 9 weeks after the clearance of viremia.^4^

A retinal lesion specific to Ebola was identified in 15% of previously symptomatic survivors.^5,6^ Lesions were predominantly nonpigmented, with a pale-gray appearance. Their shapes are variable, but sharp angulations are characteristic. Peripapillary lesions exhibited variable curvatures in keeping with the retinal nerve fiber layer projections. Ocular coherence tomography (OCT) imaging demonstrates a characteristic V-shaped hyperreflectivity of the outer nuclear layer overlying discontinuities of the ellipsoid zone and interdigitation zone in the smaller lesions, whereas larger lesions caused a collapse of the retinal layers and loss of retinal thickness. Perilesional areas of dark without pressure (DWP) (thinned ellipsoid zone hyporeflectivity) accompanied 88.7% of lesions to varying extents.

Relapses of uveitis in survivors of EVD up to 13 months after clearance of viremia have been reported (without aqueous etiological analysis),^1^ and the incidence of new uveitis at 1-year follow-up in a Liberian cohort of survivors with EVD was significantly higher vs control individuals.^7^ However, there is a high prevalence of uveitis secondary to other etiologies in West Africa,^8^ and therefore recurrent ocular inflammation in survivors of EVD may not necessarily be secondary to Ebola.^9^

This study aimed to investigate a cohort of survivors of EVD who underwent detailed ophthalmic evaluation in early 2016^5^ by undertaking reexaminations 1 year later to assess for evidence of recurrent uveitis and changes in previously identified areas of DWP.^6^

## Methods

### Study Design and Population

A prospective, controlled study of survivors of EVD who had attended the EVD survivors clinic at 34 Military Hospital in Freetown, Sierra Leone, or other medical facilities in the region and reported ophthalmic symptoms since discharge was conducted in 2016.^5^ All participants of this study who attended between January 22, 2016, and April 26, 2016, were contacted by telephone and invited to attend the ophthalmology clinic for review 1 year following their baseline examination. The study was approved by the Sierra Leone Ethics and Scientific Review Committee and followed the tenets set forth by the World Medical Association Declaration of Helsinki, seventh revision (2013).

Ebola virus disease survivor status was verified by the possession of a valid discharge certificate from an Ebola treatment unit. Positive identification of survivors of EVD at follow-up was confirmed by retinal vasculature morphology comparison. Survivors were invited to participate in English or Krio, as preferred, with local ophthalmic nurses acting as interpreters. Consent was confirmed by fingerprint or signature. Patients received treatment if required or were referred on as needed at the discretion of the examining ophthalmologist.

### Data Collection

Ophthalmic examination and imaging were obtained on all survivors of EVD, consisting of presenting and pinhole visual acuity (VA) (tumbling E-logMAR chart at 4 m); table-mounted slitlamp examination; 2WIN autorefraction (Adaptica S.r.l); color vision (14-plate Ishihara test book); icare TA01i rebound tonometry (Icare Oy); nonmydriatic, Daytona scanning laser ophthalmoscope ultra-widefield imaging (Optos PLC); and Topcon DRI Triton swept-source optical coherence tomography (OCT) (Topcon Corporation). Image analysis is described in eMethods 1 in the Supplement.

### Blood Spot Analysis

Blood spots were obtained as described in eMethods 2 in the Supplement. Blood spots (2 drops of approximately 80 μL per spot) were collected on Whatman 903 Protein saver card (GE Healthcare), passively dried at ambient room temperature (typically 25°C to 30°C), exported, and then stored at −80°C prior to analysis. *Toxoplasma gondii* immunoglobulin G (IgG) status (TOXO IgG enzyme-linked immunosorbent assay; DIAsource ImmunoAssays SA) and HIV-1 and HIV-2 status (Murex HIV-1.2.O; DiaSorin S.p.A.) were determined from the dried blood spot samples described in eMethods 3 in the Supplement.

### Main Outcome Measures

The primary outcome measure was change in retinal features on ultra-widefield imaging. Secondary outcome measures included visual impairment in survivors of EVD, surgical intervention, reoccurrence of ocular inflammation and structural complications, and detection of IgG to HIV and *T gondii*.

### Statistical Analysis

Statistical analysis was performed with SPSS, version 22 (IBM). Visual impairment was categorized using the World Health Organization’s *International Statistical Classification of Diseases and Related Health Problems, Eleventh Revision (ICD-11) *6 grade classification.^10^

## Results

Seventy-two survivors of EVD attended the 34 Military Hospital Eye Clinic, Freetown, Sierra Leone, between January 22, 2016, and April 26, 2016. One year later, of the initial 72 survivors, 12 of the survivors’ contact telephone numbers were either outside of the coverage zone or no longer available during the follow-up period (minimum of 3 communication attempts on different days). Sixty were contactable; 3 were unable to attend or declined the invitation; and 57 survivors reattended for repeated ophthalmic examination. A total of 113 of 114 eyes were examined (1 prosthetic eye). Male-to-female ratio of survivors of EVD was 1 to 1.85. Median age at the time of ophthalmic examination was 30 years (interquartile range [IQR], 25.5-39.5 years). Median time from Ebola treatment unit discharge to baseline examination was 1.09 years (397 days; IQR, 351-449.5 days).

### Visual Acuity

Comparative VA outcomes between baseline and 1-year follow-up examination are displayed in the eTable in the Supplement.

### Cataract Surgery

Three of 6 survivors of EVD diagnosed as having white cataracts (all unilateral) at baseline examination reattended for a repeated examination. One had undergone manual small-incision cataract surgery during the 1-year interim and improved VA from light perception to 0.55 logMAR. Intraocular pressure was 16 mm Hg in the operated eye. Early posterior capsular opacification limited vision in addition to an epiretinal membrane and isolated foveal neurosensory retinal detachment visible on OCT (eFigure 1 in the Supplement). A further survivor with a right dense posterior subcapsular cataract at baseline had also undergone manual small-incision cataract surgery during the study period. Visual acuity improved from hand movements to 0.7 logMAR. Fundus examination demonstrated a peripheral pigmented retinal lesion with surrounding hypopigmentation (eFigure 2 in the Supplement), not in keeping with Ebola retinopathy, suggesting a possible alternative etiology for cataract development.

### Retinal Examination

Of the 57 patients recruited, 110 of 114 eyes were amenable to retinal imaging at initial examination (no fundal view in 3 eyes secondary to cataract; 1 prosthetic eye), and 109 eyes underwent repeated imaging 1 year later (1 additional cataract limited retinal imaging). Fifteen eyes of survivors (13.6%) had retinal lesions secondary to EVD as previously identified at the initial examination,^5^ 50% of which were bilateral.

### Dark Without Pressure

**Figure 1.**
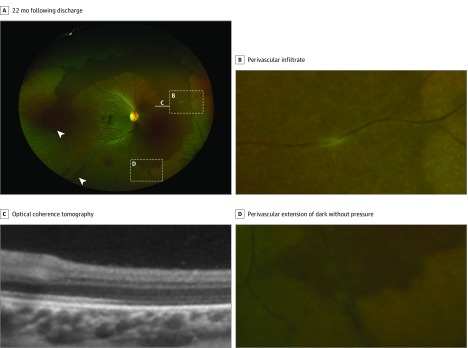
Contralateral Retinal Imaging of a Survivor With Severe Ebola Related Uveitis A, Ultra-widefield fundus image of survivor 1’s right eye. B, Perivascular infiltrate. C, OCT demonstrating thinned, hyporeflective ellipsoid zone. D, Perivascular infiltrate and extension of dark without pressure following the vascular distribution.

**Figure 2.**
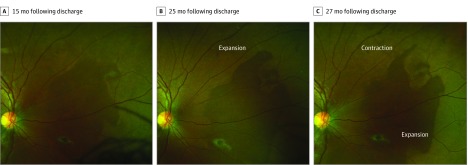
Simultaneous Expansion and Regression of Dark Without Pressure Survivor 8’s right eye. Sequential ultra-widefield fundus image comparison demonstrating simultaneous expansion and regression of dark without pressure area.

**Figure 3.**
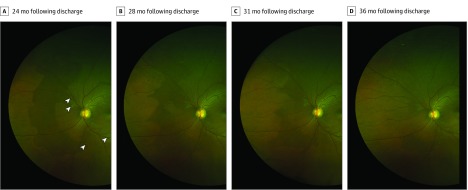
Regression of Dark Without Pressure Survivor 10’s left eye. Sequential ultra-widefield fundus image comparison demonstrating regression of dark without pressure. White arrowheads indicate Ebola retinal lesions.

**Figure 4.**
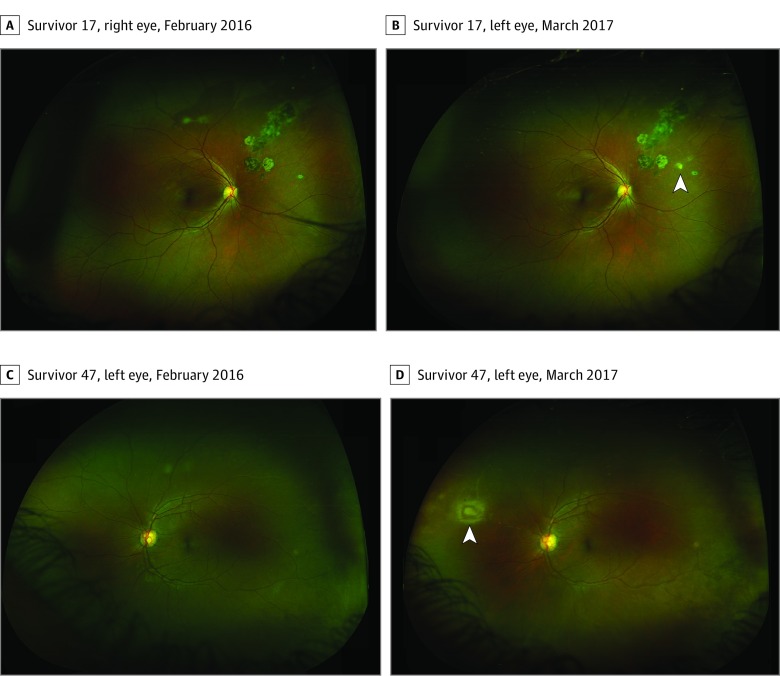
Recurrent and New Areas of Toxoplasmosis Chorioretinitis A and B, Survivor 17’s right eye (A and B), sequential ultra-widefield fundus image comparison in February 2016 (A) and March 2017 (B). White arrowhead indicates site of new retinal lesion in keeping with surrounding lesions suggestive of recurrent toxoplasmosis chorioretinitis. C and D, Survivor 47’s left eye, sequential ultra-widefield fundus image comparison in February 2016 (C) and March 2017 (D). New superior nasal retinal lesion visible in image D is in keeping with toxoplasmosis chorioretinitis and adjacent Kyrieleis vasculitis.

Perilesional DWP (<1 disc diameter extent) was observed around the circumference of EVD lesions in 7 eyes (6 survivors) and pigmented chorioretinal lesions consistent with toxoplasmosis chorioretinitis lesions in 2 eyes (2 survivors). Survivors with extensive areas of DWP, their associated features, and change during the observation period are summarized in the Table (Figures 1, 2, 3, and 4; Video; eFigures 4-9 in the Supplement). Areas of DWP were most frequently observed in the nasal, midperipheral fundus (eFigure 3 in the Supplement).

**Table.** Summary of Ebola Survivors With Extensive Dark Without Pressure and Fellow Eye Findings

Patient No.	ETU Discharge to Baseline Imaging, mo	Eye	LogMAR VA^a^	Approximate Snellen Equivalent	DWP Area			Degrees of Circumferential DWP, Quadrant/Eccentricity	DWP Type	EVD Lesions	Observations
					Baseline^b^	1 y^b^	Change, %				
2	14.9	OD	0.00	20/20	121	75.7	−37	270° T S N and 2 × Iso I areas	Iso	0	PVI with accompanying perivascular DWP extensions noted at 22 mo (Figure 1); simultaneous DWP expansion and contraction at peripheral margin; segmentation of circumferential DWP (eFigure 4 in the Supplement); shifting WWP
		OS	PL	PL	NA^c^	NA	NA	NA	NA	NA	White cataract, hypotony, seclusio-pupillae and closed angle. No fundal view (Video)
8	15.4	OD	0.00	20/20	60.3	79.1	25	90° N Enc EVD lesions, 80° I/MP	ExPL, Iso	3	Simultaneous DWP expansion and contraction at N peripheral margin; shifting WWP (Figure 2)
		OS	0.00	20/20	NA	NA	NA	NA	NA	0	Shifting WWP
10	24.1	OD	0.1	20/25	NA	NA	NA	DWP confined to EVD lesion margins	PL	4	Minimal change
		OS	0.1	20/25	38.4	7.8	−80	180° N Enc lesions/MP	ExPL	3	Predominant peripheral DWP resolution, with scalloped edge formation and segmentation of circumferential DWP (Figure 3); T WWP coalesce
17	13.6	OD	0.2	20/32	44.6	33.3	−25	S N Enc lesions/MP, FP	ExPL	0	Pigmented chorioretinal lesions. New satellite lesion with mild vitritis (Figure 4A and B)
		OS	0.14	20/25	NA	NA	NA	DWP confined to non–Ebola lesion margin	DWP confined to non–Ebola lesion margin	DWP confined to non–Ebola lesion margin	I T pigmented chorioretinal lesion
20	4	OD	0.06	20/25	6.4	13.6	113	Confined to lesion margins	ExPL	0	Pigmented chorioretinal lesion adjacent to the optic disc (eFigure 5 in the Supplement)
		OS	0.06	20/25	NA^c^	NA	NA	NA	NA	0	2 pigmented lesions <1/4 DD
24	14.3	OD	0.00	20/20	NA^c^	NA	NA	NA	NA	0	Normal fundus
		OS	0.3	20/40	201.9	73.7	−26^c^	330° T N S/MP FP	Iso	0	Circumferential DWP reduced to 315° at 1 y and reduced DWP peripheral extension
26	12.7	OD	0.1	20/25	NA^c^	NA	NA	DWP confined to non-Ebola lesion margin	PL	0	ST FP pigmented chorioretinal lesion
		OS	0.12	20/25	9.7	17.8	84	56° N/MP	Iso	0	Peripheral DWP expansion (eFigure 6 in the Supplement)
34	13.7	OD	0.1	20/25	59.2	81.0	+37^d^	MP	ExPL	12	Extensive DWP, boundary ill defined; DWP peripheral expansion; WWP coalesce
		OS	0.06	20/25	55.2	62	+12^d^	MP	ExPL	10	DWP peripheral expansion; shifting WWP (eFigure 7 in the Supplement)
38	15.1	OD	0.3	20/40	246	190.7	+18^d^	360° MP, FP	Iso	0	Circumferential DWP expansion (eFigure 8 in the Supplement)
		OS	0.5	20/63	171.4	207.4	+21^d^	230° N I/MP, FP	Iso	0	DWP expansion into beyond visible retina on imaging (eFigure 9 in the Supplement); temporal WWP expansion
56	13.7	OD	0.06	20/25	1.86	0	−100	S 3 × Iso areas MP, FP	Iso	0	DWP resolution at 1 y
		OS	0.08	20/25	NA^c^	NA	NA	NA	NA	0	Normal fundus

Abbreviations: DD, disc diameter; DWP, dark without pressure; ETU, Ebola treatment unit; EVD, Ebola virus disease; ExPL, extensive DWP encompassing lesions; FP, FarPeriphery; I, inferior fundus; Iso, isolated (not associated with other retinal lesions); MP, midperiphery; N, nasal fundus; NA, not applicable; Pig, pigmented; PL, perilesional; PVI, perivascular infiltrates; S, superior fundus; T, temporal fundus; VA, visual acuity; WWP, white without pressure.

^a^ Presenting logMAR at 1-year follow-up examination.

^b^ Area measured in optic disc areas.

^c^ No DWP.

^d^ DWP margins only partly defined. Calculated area represents comparable defined DWP boundary. Area represents an underestimation of true DWP extent.

**Video.** Anterior Chamber Biomicroscopy in a Patient With Ebola Virus Disease This slitlamp examination video illustrates left eye anterior chamber abnormalities consistent with previous severe inflammation, including residua of a white cataract (1), synechiae between the iris and posterior chamber (4), and a narrow angle between the cornea (3) and iris (2) in an eye with very low intraocular pressure (hypotonous) without active inflammation.

No intraocular inflammation was associated with areas of DWP. No new retinal lesions in keeping with EVD were observed in survivors with or without previously identified Ebola retinal lesions during the follow-up period. New retinal lesions in keeping with toxoplasmosis chorioretinitis were observed in 2 survivors of EVD during the follow-up period (Figure 4) and were associated with mild vitritis.

### Immunological Analysis

Sufficient blood spot samples were obtained from 56 of 57 survivors studied to enable an analysis of both *T gondii* IgG and HIV IgG status. Forty-one survivors (73%) were positive for *T gondii* IgG, and 1 (2%) was HIV IgG positive within this cohort.

## Discussion

We compared the ocular findings of a cohort of 57 Sierra Leonean survivors of the 2014 to 2016 Ebola epidemic between a year and 2 years following discharge. We did not find any evidence of recurrent EVD retinal lesions during this period. However, we provide widefield fundus imaging comparisons to demonstrate active progression and regression of areas of DWP, which was associated with transient perivascular infiltrates in 1 survivor. Dark without pressure was present in association with Ebola retinal lesions, with retinal lesions of other etiology, and in isolation. We also report the occurrence of new clinical toxoplasmosis chorioretinitis in 2 survivors of EVD within the period of observation, and the outcomes of survivors who have undergone cataract surgery during this time.

### Dark Without Pressure

Isolated areas of homogeneous, geographical, flat, brown areas of the fundi were first described as DWP in 1975 by Nagpal et al.^11^ Unlike the name suggests, no association with or without pressure/ocular indentation has been reported. Despite the duration since its initial observation, DWP has attracted limited interest in the scientific literature and remains poorly understood.^6,12,13,14,15^ There remains no histological correlation. We previously demonstrated that DWP correlates to a thinned, hyporeflective second outer retinal band and a reflective loss of the third hyperreflective band on OCT,^6^ termed the *ellipsoid zone (EZ)* and *interdigitation zone*, respectively, by current consensus.^16^ This led us to the hypothesis that DWP is caused by a change in mitochondrial state. Cellular characterization using immunohistochemistry markers^17^ concur with Spaide and Curcio^18^ that the EZ is generated by the photoreceptor inner segment ellipsoids, secondary to the tight bundles of mitochondria^18,19,20,21,22^ as opposed to the *inner segment/outer segment* junction as previously termed.^23,24^ In vitro, the refractivity of isolated mitochondria is affected by their metabolic state.^25,26^ Although retinal functional assessment of areas of DWP is limited, attenuation of the EZ on OCT is well recognized as a detrimental sign of cell function and health,^27^ and the restoration of this band has been associated with restored VA following retinal detachment surgery^28^ and macula hole closure.^29^

The sequential imaging in this study has provided new insights into the behavior of these areas. Contraction and expansion of areas of DWP have been previously observed^11,15^; however, Figure 2 and eFigure 3 in the Supplement provide evidence that affected areas of DWP do not behave in a unified manner, ie, equally expanding or contracting in unison. Instead, while one border may advance to occupy further retinal territory, neighboring borders can simultaneously retract.

Expansion of areas of DWP appears to occur contiguously from the margin of existing areas. Similarly, resolution of areas of DWP only occur from their peripheral margins and do so at spatially uneven velocities, often leading to the formation of scalloped edges (Figure 3) that can advance sufficiently through an area to create segmentation and isolated areas of DWP (Figure 3; eFigure 3 in the Supplement).

Where areas of DWP appear spatially associated with Ebola retinal lesions, regression appears to occur toward the retinal lesion (Figure 3), leaving a persistent perilesional halo around most lesions. This raises the possibility that where only perilesional areas of DWP were identified at baseline imaging (a year following Ebola treatment unit discharge), more extensive areas of DWP may have been present at an earlier period during convalescence.

No signs of intravitreal or retinal inflammation were observed in survivors without new clinical toxoplasmosis chorioretinitis, except for 1 survivor where multifocal perivascular infiltrates were observed. These were within areas of DWP (with no visible Ebola retinal lesions), with fingerlike extensions of DWP seen following the affected vessels at 22 weeks following discharge (Figure 1A and D).

The presence of DWP around Ebola retinal lesions, which retracts back toward Ebola retinal lesions while demonstrating ongoing fluctuations of expansion and contraction, strongly suggests the presence of an ongoing intraretinal stimulus associated with the Ebola retinal lesion. Whether this represents ongoing intraretinal viral persistence remains speculative, but reports of viral RNA persistence up to 40 months following symptom onset in semen samples^7^ suggest it is theoretically plausible. Dark without pressure is not specific to Ebola, so maybe a nonspecific response to infection or triggered by infection. Dark without pressure is easier to discern in pigmented fundi and is therefore perhaps more widespread in white or lightly pigmented fundi than realized.

### Cataracts

In our primary cohort (n = 82), white cataracts only occurred unilaterally (n = 6) with normal VA in the contralateral eye. There is increasing evidence to suggest cataract surgery can be safely conducted in survivors of EVD.^30^ All reported reverse-transcriptase polymerase chain reaction test results of aqueous fluid in survivors of EVD with recurrent uveitis^31^ or before cataract surgery^5,30^ have been negative for Ebola virus. Cataract surgery performed on survivors of EVD within our cohort demonstrated VA improvements; however, early posterior capsular opacification limited maximal visual benefit, and in-country access to Nd:YAG laser facilities are very limited. New retinal lesions indicative of toxoplasmosis chorioretinitis were visible on the fundus of 1 survivor within this cohort following small-incision cataract surgery for posterior subcapsular cataracts. The seroprevalence of *T gondii* IgG was 73% within our cohort, in keeping with uveitis secondary to *T gondii* being common in West Africa.^8^ Retinal lesions suggestive of toxoplasmosis chorioretinitis were also present in 18.2% and 19% of survivors of EVD and local control individuals, respectively, in our previous study.^5^ Therefore, a differential diagnosis for cataracts within survivors of EVD must include alternative etiologies that are common in the West African population.

### Evidence of Recurrence

Recurrent episodes of uveitis in survivors of EVD in Guinea have been attributed to Ebola without polymerase chain reaction corroboration.^1^ The detection of new retinal lesions with the typical appearance of toxoplasmosis chorioretinitis in 2 survivors (4%) of this cohort during a 1-year observation period suggests any uveitis recurrence in survivors of EVD in West Africa may be owing to an alternative etiology, although further confirmatory serological or aqueous analysis was not conducted in this study. Current World Health Organization guidelines for the management of uveitis in survivors of EVD suggest systemic corticosteroids (adults) or methotrexate (children) if no resolution is seen within 7 days of topical prednisolone.^32^ The high prevalence of toxoplasmosis within the region is recognized^8^ and within this cohort was identified by serology, together with clinical evidence suggestive of recurrent toxoplasmosis chorioretinitis. When faced with episodes of recurrent, pan or posterior uveitis in survivors of EVD, clinicians should consider *T gondii* chorioretinitis in the differential diagnosis. If *T gondii* cannot be excluded, appropriate treatment should be included in the treatment plan.

### Limitations

Our study has limitations in part related to the setting, with severely limited health care infrastructure. The prevalence of DWP within the general population in West Africa is currently unknown. Our cohort was drawn from survivors of EVD who previously reported ocular symptoms; therefore, generalizations to the survivor population as a whole are uncertain. Most patients were examined a year apart, so the occurrence of asymptomatic uveitis episodes or reversible changes to lesions in the intervening time is unknown. Dimension calculations of peripheral lesions on widefield imaging are susceptible to underestimation owing to peripheral warping in projecting a 3-dimensional retina to a 2-dimensional image.

## Conclusions

No new cases of recurrent uveitis secondary to Ebola were identified during the 1-year observation period within a cohort of 57 survivors, and VA was maintained. New chorioretinal lesions typical of *T gondii* chorioretinitis were identified. Dark without pressure both enlarged and contracted. Although the relevance of DWP is undetermined, our observations are consistent with a retinal response to an infectious etiology possibly through mitochondrial change. Cataract secondary to EVD uveitis was uncommon and unilateral. Cataract surgery appears to be safe and can enable visual gains.

## Notes

Supplement. **eTable.** Comparative ebola survivor best and worst eye visual acuity **eMethods 1. **Quantitative image analysis **eMethods 2. **Dried blood spot collection protocol **eMethods 3. **Dried blood spot analysis **eFigure 1. **Survivor No.25 Right eye, post-cataract surgery macular OCT **eFigure 2. **Survivor No.32 Right eye, ultra-widefield fundus image **eFigure 3. **Dark-without- pressure retinal distribution heat map **eFigure 4. **Survivor No.2 Right eye, sequential fundus image comparison **eFigure 5. **Survivor No.20 Right eye, sequential fundus image comparison **eFigure 6. **Survivor No.26 Left eye, ultra-widefield fundus image comparison **eFigure 7. **Survivor No.34 Left eye, sequential fundus image comparison **eFigure 8.** Survivor No.38 Right eye, sequential fundus image comparison **eFigure 9. **Survivor No.38 Left eye, sequential fundus image comparison
