# Supplementary material for: Evolving Longitudinal Retinal Observations in a Cohort of Survivors of Ebola Virus Disease
Source: JAMA Ophthalmol. 2020 Mar 5;138(4):395–403. doi: 10.1001/jamaophthalmol.2020.0173 (PMC7146102; doi:10.1001/jamaophthalmol.2020.0173)
Supplement: Supplement. — eTable. Comparative ebola survivor best and worst eye visual acuity eMethods 1. Quantitative image analysis eMethods 2. Dried blood spot collection protocol eMethods 3. Dried blood spot analysis eFigure 1. Survivor No.25 Right eye, post-cataract surgery macular OCT eFigure 2. Survivor No.32 Right eye, ultra-widefield fundus image eFigure 3. Dark-without- pressure retinal distribution heat map eFigure 4. Survivor No.2 Right eye, sequential fundus image comparison eFigure 5. Survivor No.20 Right eye, sequential fundus image comparison eFigure 6. Survivor No.26 Left eye, ultra-widefield fundus image comparison eFigure 7. Survivor No.34 Left eye, sequential fundus image comparison eFigure 8. Survivor No.38 Right eye, sequential fundus image comparison eFigure 9. Survivor No.38 Left eye, sequential fundus image comparison [file jamaophthalmol-138-395-s001.pdf]

Supplementary Online Content

Steptoe PJ, Momorie F, Fornah AD, et al. Evolving longitudinal retinal observations in a cohort of survivors of ebola virus disease. *JAMA Ophthalmol*. Published online March 5, 2020. doi:10.1001/jamaophthalmol.2020.0173

eTable 1. Comparative ebola survivor best and worst eye visual acuity
eMethods 1. Quantitative image analysis
eMethods 2. Dried blood spot collection protocol
eMethods 3. Dried blood spot analysis
eFigure 1. Survivor No.25 Right eye, post-cataract surgery macular OCT
eFigure 2. Survivor No.32 Right eye, ultra-widefield fundus image
eFigure 3. Dark-without- pressure retinal distribution heat map
eFigure 4. Survivor No.2 Right eye, sequential fundus image comparison
eFigure 5. Survivor No.20 Right eye, sequential fundus image comparison
eFigure 6. Survivor No.26 Left eye, ultra-widefield fundus image comparison
eFigure 7. Survivor No.34 Left eye, sequential fundus image comparison
eFigure 8. Survivor No.38 Right eye, sequential fundus image comparison
eFigure 9. Survivor No.38 Left eye, sequential fundus image comparison

This supplementary material has been provided by the authors to give readers additional information about their work.

© 2020 Steptoe PJ et al. *JAMA Ophthalmol.*

eTable 1. Comparative Ebola Survivor Best and Worst Eye Visual Acuity

**WHO Classification**[10]**,** [Category], Snellen Visual Acuity	**Best Eye**		**Worse Eye**	
	**Baseline**	**1 Year** [a]	**Baseline**	**1 Year** [a]
[0] No visual impairment<br>≥ 20/40, eyes [%]	52 [91%]	53 [93%]	44 [77%]	45 [79%]
[1] Mild vision impairment<br>< 20/40 AND ≥ 20/70, eyes [%]	2 [4%]	2 [4%]	2 [4%]	3 [5%]
[2] Moderate vision impairment<br>< 20/70 AND ≥ 20/200, eyes [%]	1 [2%]	2 [4%]	5 [9%]	5 [9%]
[3] Severe vision impairment<br>< 20/200 AND ≥ 20/400, eyes [%]	0 [0%]	0 [0%]	0 [0%]	0 [0%]
[4] Blindness<br>< 20/400 AND ≥ 20/1200, eyes [%]	0 [0%]	0 [0%]	0 [0%]	0 [0%]
[5] Blindness<br>< 20/1200 AND ≥ Light perception, eyes [%]	0 [0%]	0 [0%]	3 [5%]	3 [5%] [b]
[6] Blindness<br>No perception of light, eyes [%]	0 [0%]	0 [0%]	0 [0%]	0 [0%]
[9] Undetermined/unspecified, eyes [%]	1 [2%] [c]	0 [0%]	1 [2%] [c]	0 [0%]
Ocular prosthesis, eyes [%]	0 [0%]	0 [0%]	1 [2%] [d]	1 [2%]
Missing Data, eyes [%]	1 [2%]	0 [0%]	1 [2%]	0 [0%]

[a] Median interval between first and last examination 370 days (IQR 365-397.5). Last examination median 2.13 years (779 days, IQR 732-821 days) from ETU discharge.

[b] Secondary to unilateral white cataracts (2 eyes), and primary open angle glaucoma in combination with significant nuclear sclerotic cataract (1 eye).

[c] Survivors age prevented Snellen VA assessment. Fixing and following acuity recorded

[d] Enucleation and prosthesis secondary to unrelated incident prior to Ebola infection.

© 2020 Steptoe PJ et al. *JAMA Ophthalmol.*

eMethods 1: Quantitative Image analysis

- Dark-without-pressure area quantification
    - Optomap® images were exported to ImageJ (version 10.2). Optic nerve area measured. Dark-without-pressure boundary was manually delineated and area calculated divided by the optic disc area.
- Frequency of dark-without pressure heat distribution map
    - A retinal grid (eFigure 9) was digitally placed over the fundus using Affinity designer (version 1.6.1). The centre of the optic disc and fovea were horizontally aligned. Each grid space was divided proportionally into ten depending on the extent of dark-without-pressure occupying the space. All imaging which included distinguishable margins of extensive areas of dark-without-pressure were included in the analysis.

© 2020 Steptoe PJ et al. *JAMA Ophthalmol.*

eMethods 2: Blood Spot Collection Protocol

1. Name and date entered on the Whatman® 903 protein saver card
2. Disposable latex rubber gloves donned.
3. Patient hands warmed. A chosen finger is massaged anterogradely to increase blood flow towards to puncture site.
4. The skin of the palmar side of the tip distal phalanx of the third or fourth finger cleaned with 70% isopropyl alcohol. Skin punctured with a single-use Accu-Chek® Safe-T-Pro Plus lancet. The finger is then held in such a position that gravity facilitates the collection of blood on the fingertip.
5. A single drop of blood delivered to 2 spots of the Whatman® protein saver card
6. When the collection of capillary blood by skin puncture is complete, cotton wool is placed on the fingertip until the bleeding has stopped.
7. Allow the protein saver card to dry at room temperature.
8. Once dried, transfer the protein saver card to a ziplock bag with a desiccant pouch enclosed.
9. Samples stored at -80°c until transported to the UK for analysis.

© 2020 Steptoe PJ et al. *JAMA Ophthalmol.*

eMethods 3: Protein Saver Card Analysis

- A 6mm diameter complete circle of dried blood spot was punched from the card and extracted overnight at 4°C in 230µl DBS elution buffer (PBS/0.05% Tween 20/0.08% sodium azide).

- For the TOXO IgG assay, eluates were diluted 1 in 100 in DBS buffer before following the Toxo IgG ELISA (KAPDTOXOG - DiaSource) standard kit protocol.

- For the HIV assay eluates were not diluted. The standard HIV-1.2.O Kit (9E25-01 - Murex) protocol was followed

- Absorbance was measured using a POLARstar omega microplate reader and analysed with MARS software (BMG Labtech).

- Positivity was determined by a standard curve for Toxo IgG and the cut-offs stipulated in the kit protocol for HIV.

© 2020 Steptoe PJ et al. *JAMA Ophthalmol.*

eFigure 1: Survivor No.25 Right Eye, swept-source OCT demonstrating an epiretinal membrane and isolated foveal neurosensory retinal detachment seen following cataract surgery.

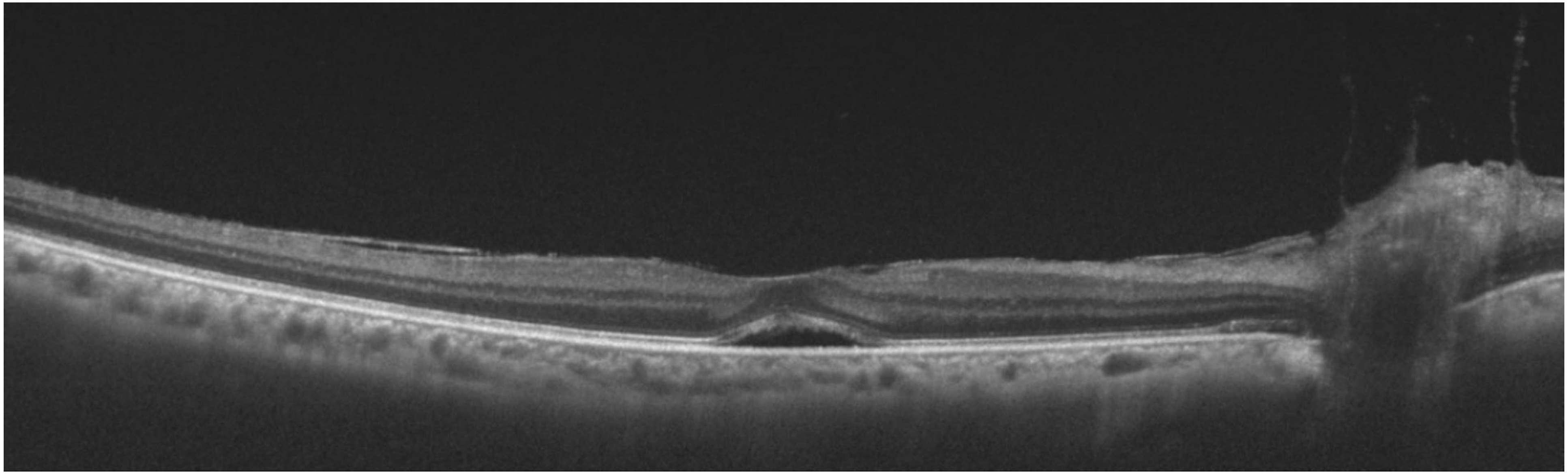

© 2020 Steptoe PJ et al. *JAMA Ophthalmol.*

eFigure 2: Survivor No.32 Right eye, scanning laser ophthalmoscope fundus image following cataract surgery. White arrow indicates pigmented retinal lesion with gray perilesional halo.

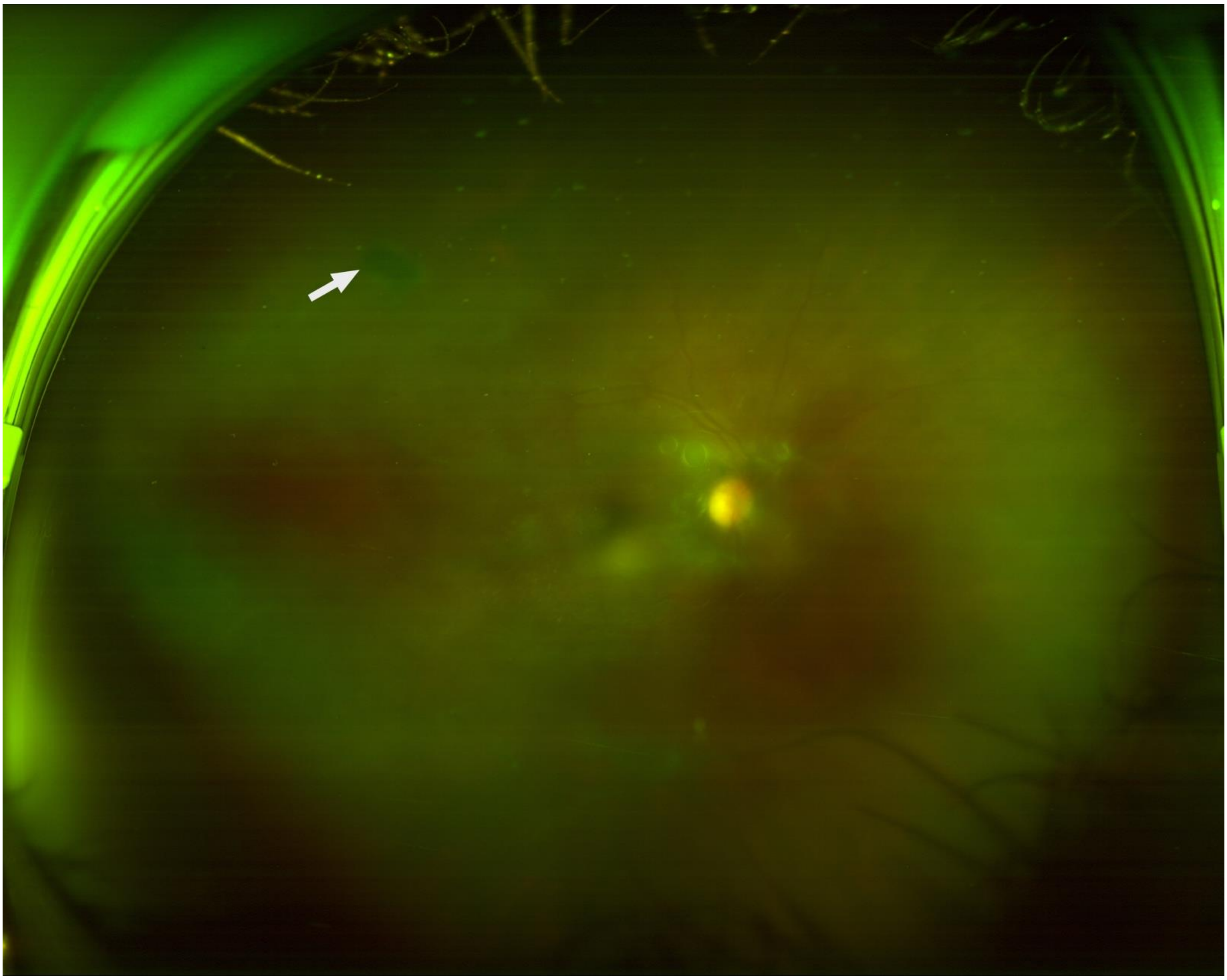

© 2020 Steptoe PJ et al. *JAMA Ophthalmol.*

eFigure 4: Survivor No.2 Right eye, sequential ultra-widefield fundus image comparison demonstrates a gradual reduction in the area of dark-without-pressure during the period of observation. Asterisk on the fundal image at 33 months post-discharge demonstrates an area of localised expansion despite overall reducing trend.

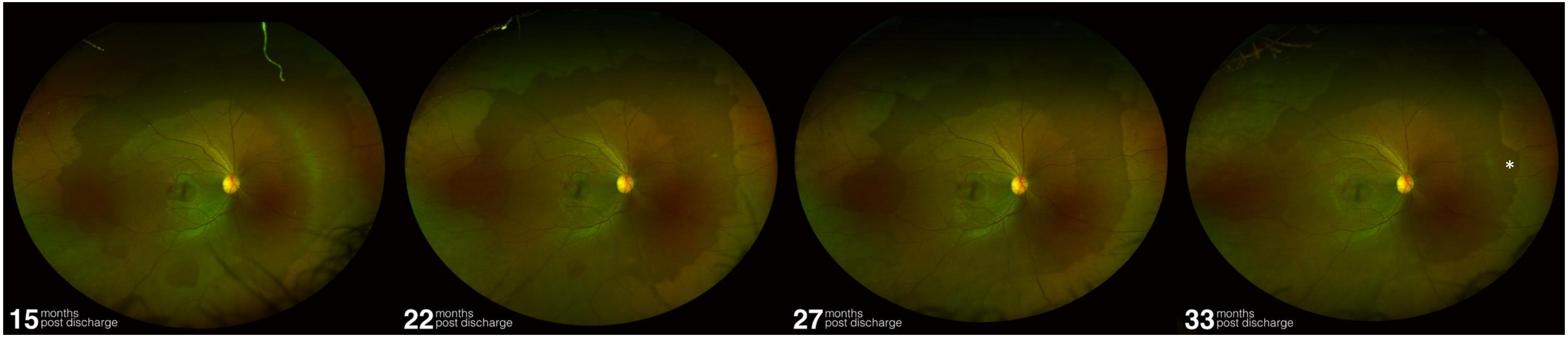

© 2020 Steptoe PJ et al. *JAMA Ophthalmol.*

eFigure 5: Survivor No. 20 Right eye, sequential ultra-widefield fundus image comparison. Shifting area of dark-without-pressure over 12 months.

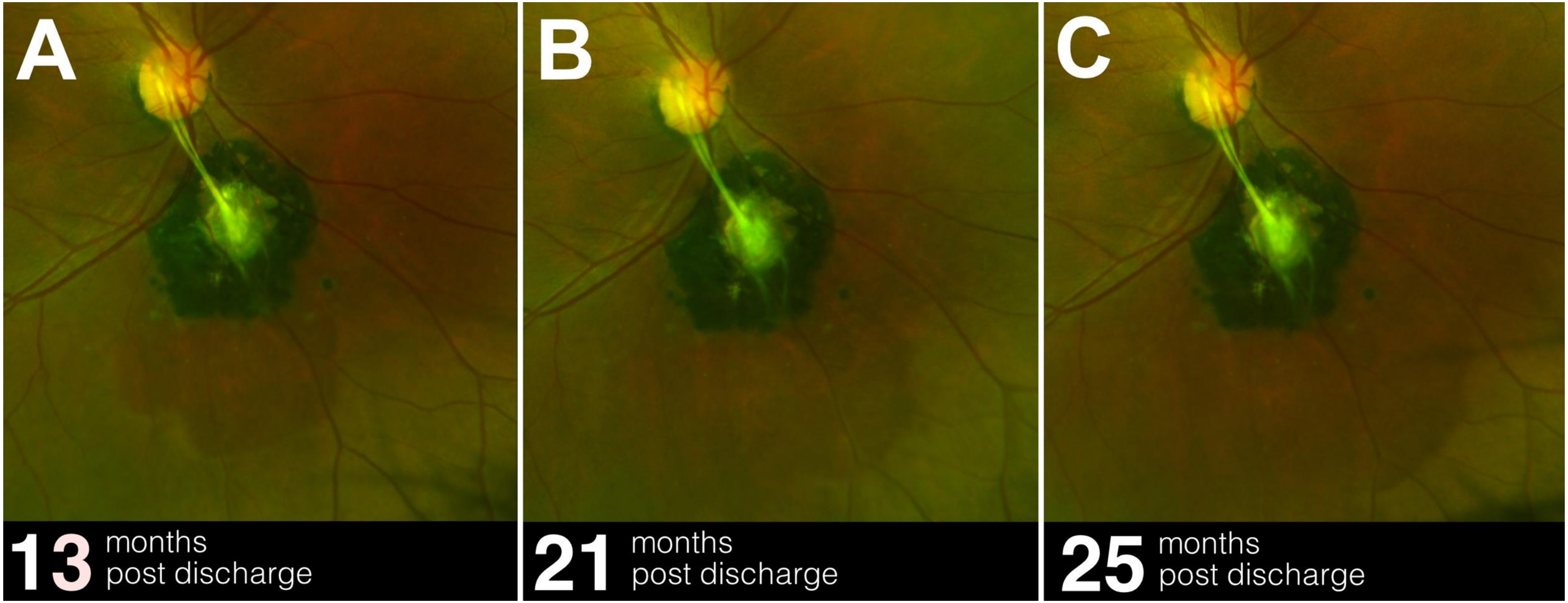

© 2020 Steptoe PJ et al. *JAMA Ophthalmol.*

eFigure 6: Survivor No.26 Left eye, sequential ultra-widefield fundus image comparison.

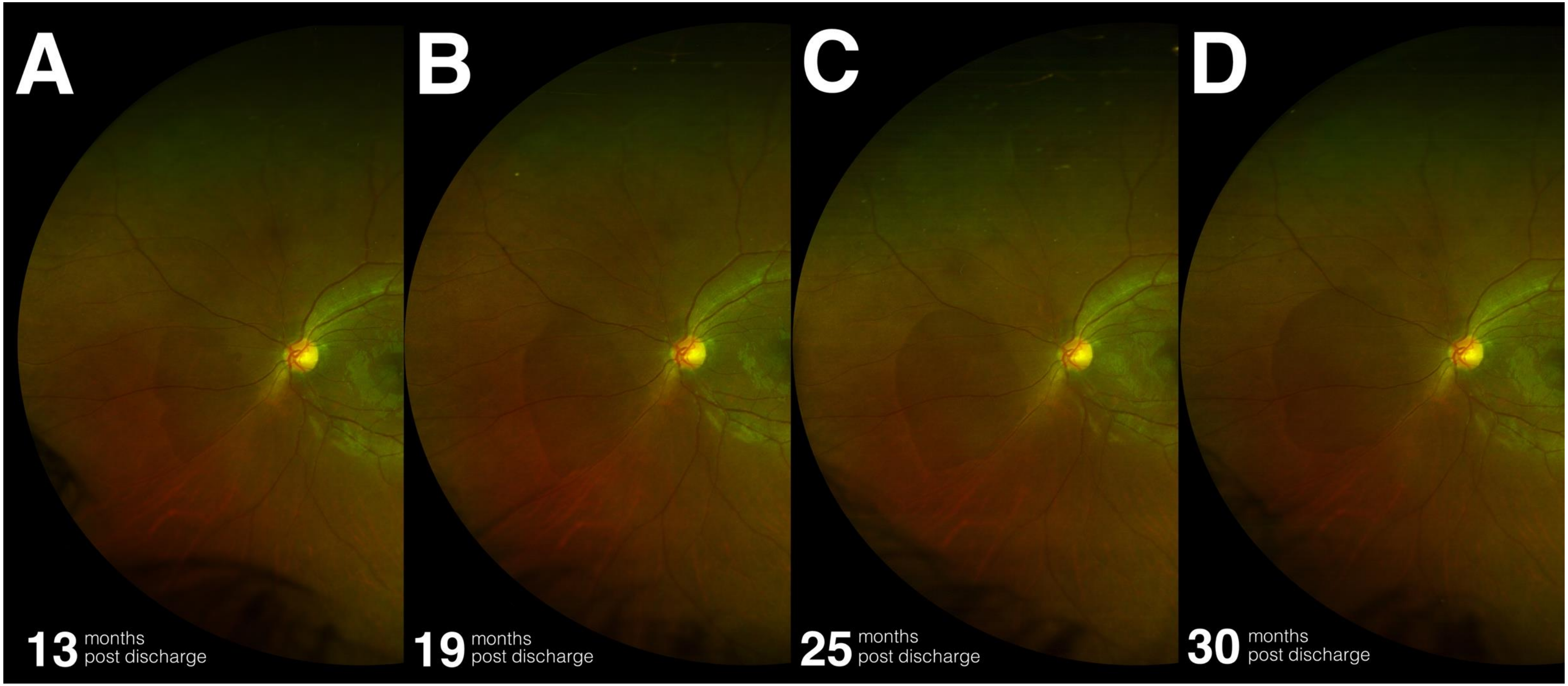

© 2020 Steptoe PJ et al. *JAMA Ophthalmol.*

© 2020 Steptoe PJ et al. *JAMA Ophthalmol.*

eFigure 7: Survivor No.34 Left eye, sequential ultra-widefield fundus comparison. Expanding border of dark-without-pressure seen advancing throughout the observation period. Image C captured utilising eye steering to permitting a greater peripheral view where an area of white-without-pressure is also visible. Dotted line in C indicates the extent of peripheral view with central fixation as in image A and B.

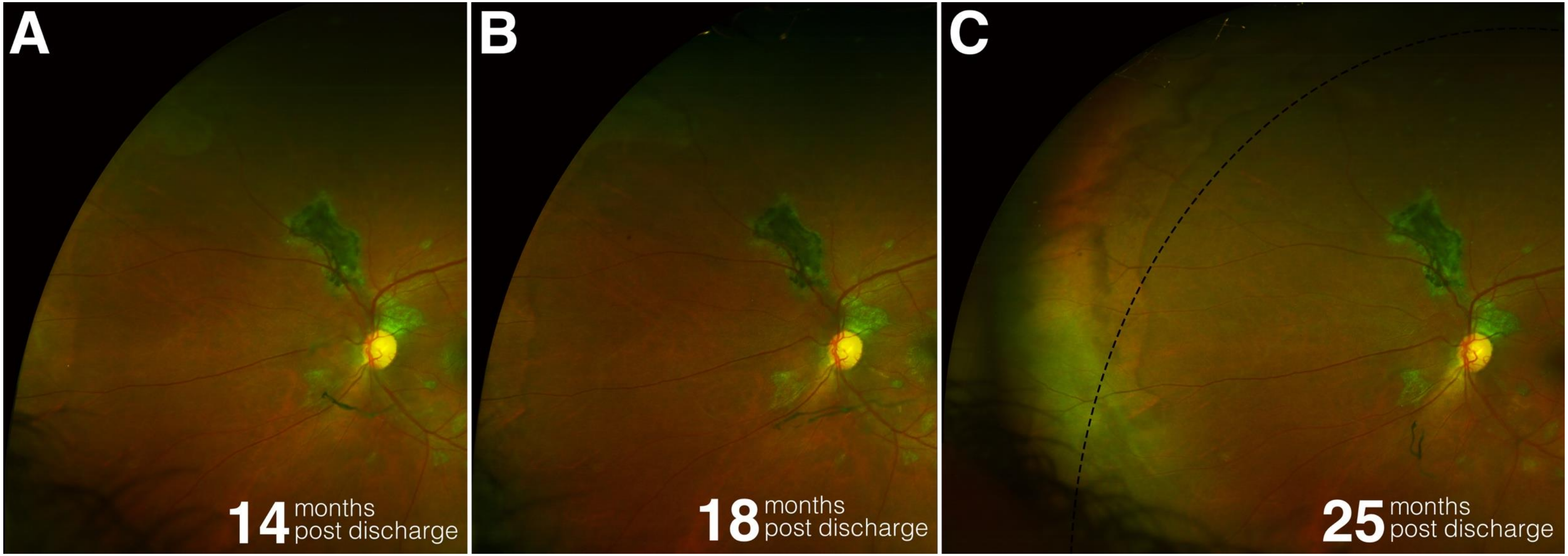

© 2020 Steptoe PJ et al. *JAMA Ophthalmol.*

eFigure 8: Survivor No.38 Right eye, sequential ultra-widefield fundus image comparison. Inferior images display an enlarged view of the inferior nasal margin of an area of enlarging dark-without-pressure. Retinal vein highlighted in black to enable spatial comparison.

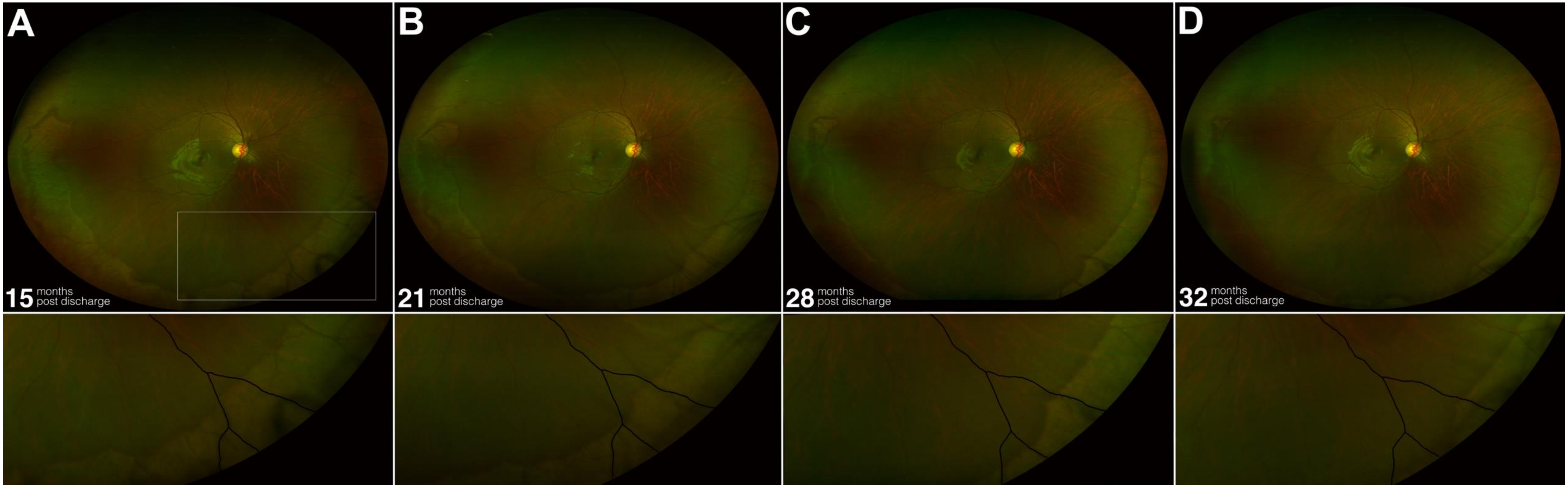

© 2020 Steptoe PJ et al. *JAMA Ophthalmol.*

eFigure 9: Survivor No.38 Left eye, sequential ultra-widefield fundus image comparison. Inferior imaging displays an enlarged view of the superior-nasal margin of enlarging dark-without-pressure.

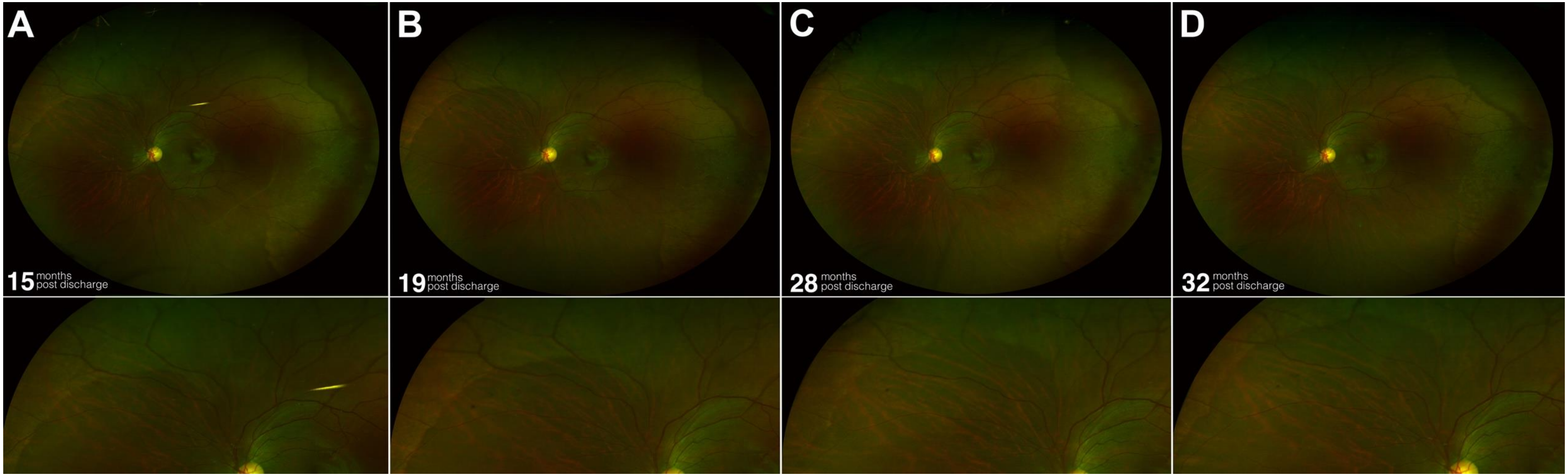

© 2020 Steptoe PJ et al. *JAMA Ophthalmol.*

eFigure 3: Dark Without Pressure retinal distribution heat map

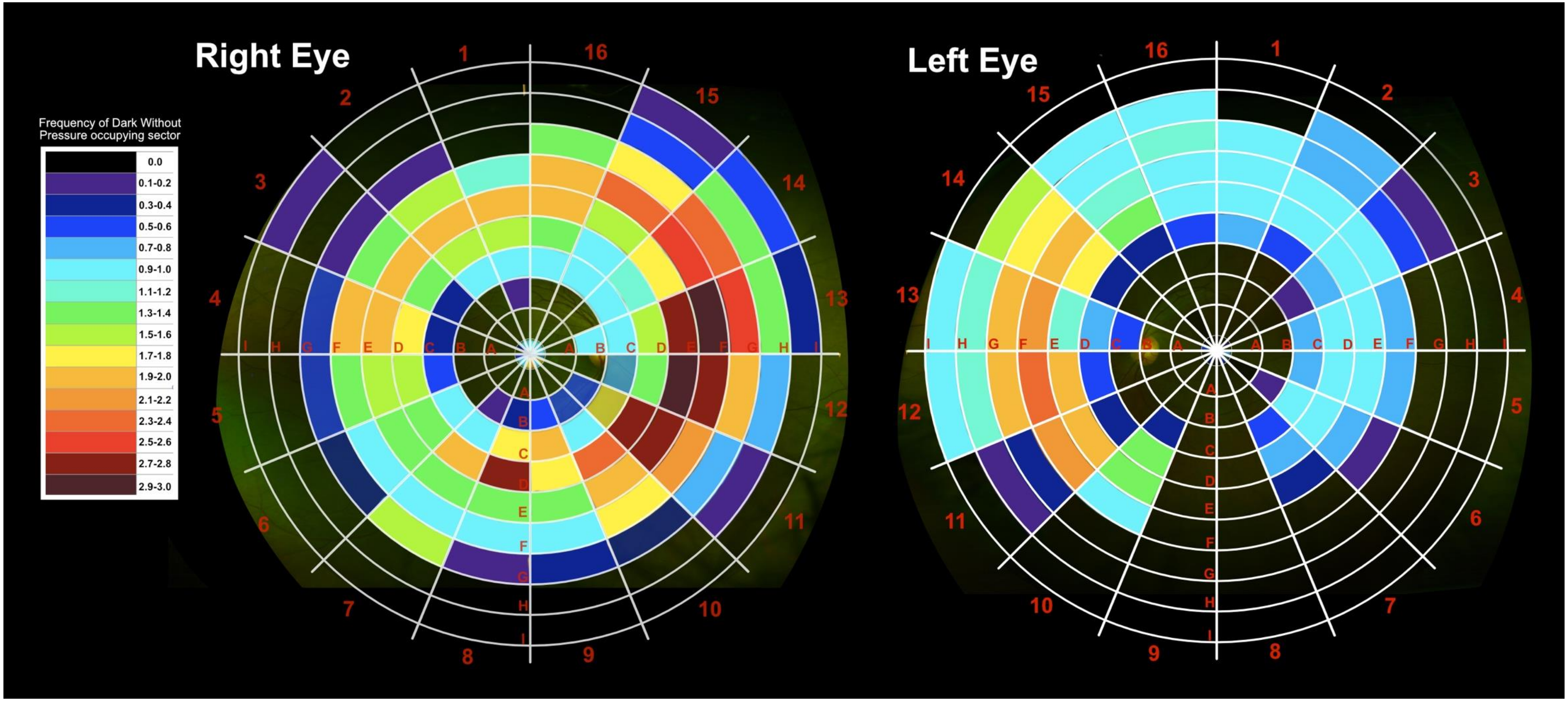

© 2020 Steptoe PJ et al. *JAMA Ophthalmol.*
